# Supplementary material for: Single molecule tracking and analysis framework including theory-predicted parameter settings
Source: Sci Rep. 2021 May 4;11:9465. doi: 10.1038/s41598-021-88802-7 (PMC8096815; doi:10.1038/s41598-021-88802-7)
Supplement: Supplementary file 1 — Supplementary Information [file 41598_2021_88802_MOESM1_ESM.pdf]

Supplementary information

Single molecule tracking and analysis framework including theory-predicted parameter settings

Timo Kuhn [1,*], Johannes Hettich[1,*], Rubina Davtyan[1,2] and J. Christof M. Gebhardt[1,#]

[1] Institute of Biophysics, Ulm University, Albert-Einstein-Allee 11, 89081 Ulm, Germany
[2] current adress: NanoLund and Solid State Physics, Lund University, Box 118, SE-22100 Lund, Sweden
[*] These authors contributed equally
# To whom correspondence should be addressed. Email: christof.gebhardt@uni-ulm.de

TrackIt manual

Release 1.1

written by

Timo Kuhn and Johannes Hettich

Gebhardt lab, University of Ulm

March 2021

Contents

1 General Information **1**

1.1 Licence . . . 1

1.2 Requirements . . . 1

1.3 Installation . . . 1

2 Workflow **2**

2.1 Overview of the main GUI . . . 2

2.2 Import movies using movie selector . . . 4

2.3 Load reference channel . . . 5

2.4 Region of interest . . . 7

2.5 Tracking . . . 7

2.5.1 Spot detection and tracking parameters . . . 7

2.5.2 Tracking routine . . . 9

2.5.3 Automatic determination of tracking parameters . . . 9

2.6 Visualization of tracks . . . 10

2.6.1 Plotting options . . . 10

2.6.2 Track explorer . . . 11

2.6.3 Detection mapping and jump distance mapping . . . 14

2.7 Data analysis tool . . . 14

2.7.1 Overview . . . 16

2.7.2 Mobility analysis tab . . . 18

2.7.3 Tracked fraction analysis tab . . . 20

2.7.4 Statistics tab . . . 22

2.8 Analysis of dissociation rates with GRID . . . 23

2.9 Data export and movie creation . . . 23

2.10 Additional tools . . . 24

2.10.1 Spot statistics . . . 24

2.10.2 Kymograph . . . 26

2.10.3 Movie splitter . . . 27

3 Benchmark **28**
3.1 Tracking performance 28
3.2 Computation times 30
3.3 Diffusion coefficient analysis 32

1 General Information

1.1 Licence

This program is free software: you can redistribute it and/or modify it under the terms of the GNU General Public License as published by the Free Software Foundation, either version 3 of the License, or (at your option) any later version. This program is distributed in the hope that it will be useful, but WITHOUT ANY WARRANTY; without even the implied warranty of MERCHANTABILITY or FITNESS FOR A PARTICULAR PURPOSE. See the GNU General Public License for more details. You should have received a copy of the GNU General Public License along with this program. If not, see `http://www.gnu.org/licenses/`.

1.2 Requirements

- Operating system: Windows 10 64-bit
- Matlab version 2017b and above
- For full functionality, the following Matlab toolboxes are required: Optimization, Image Processing, Statistics and Machine Learning, Parallel Computing

1.3 Installation

1. TrackIt is available from `https://gitlab.com/GebhardtLab/TrackIt`
2. Extract
3. Install the GRID toolbox ("GRID_for_trackit.mltbx") that comes with TrackIt.
4. Open the TrackIt_v1_1.m file with Matlab
5. Run TrackIt by clicking the run button (F5) or from the command line

2 Workflow

2.1 Overview of the main GUI

TrackIt is a tracking and analysis pipeline for single-molecule fluorescence microscopy movies. The Software uses a unique batch data structure to handle and save all data created by the user, it can be stored in a .mat file and loaded into the software at any point of the workflow. Only one .mat file is needed per data-set which makes it easy to analyze and compare many sets of experiments.

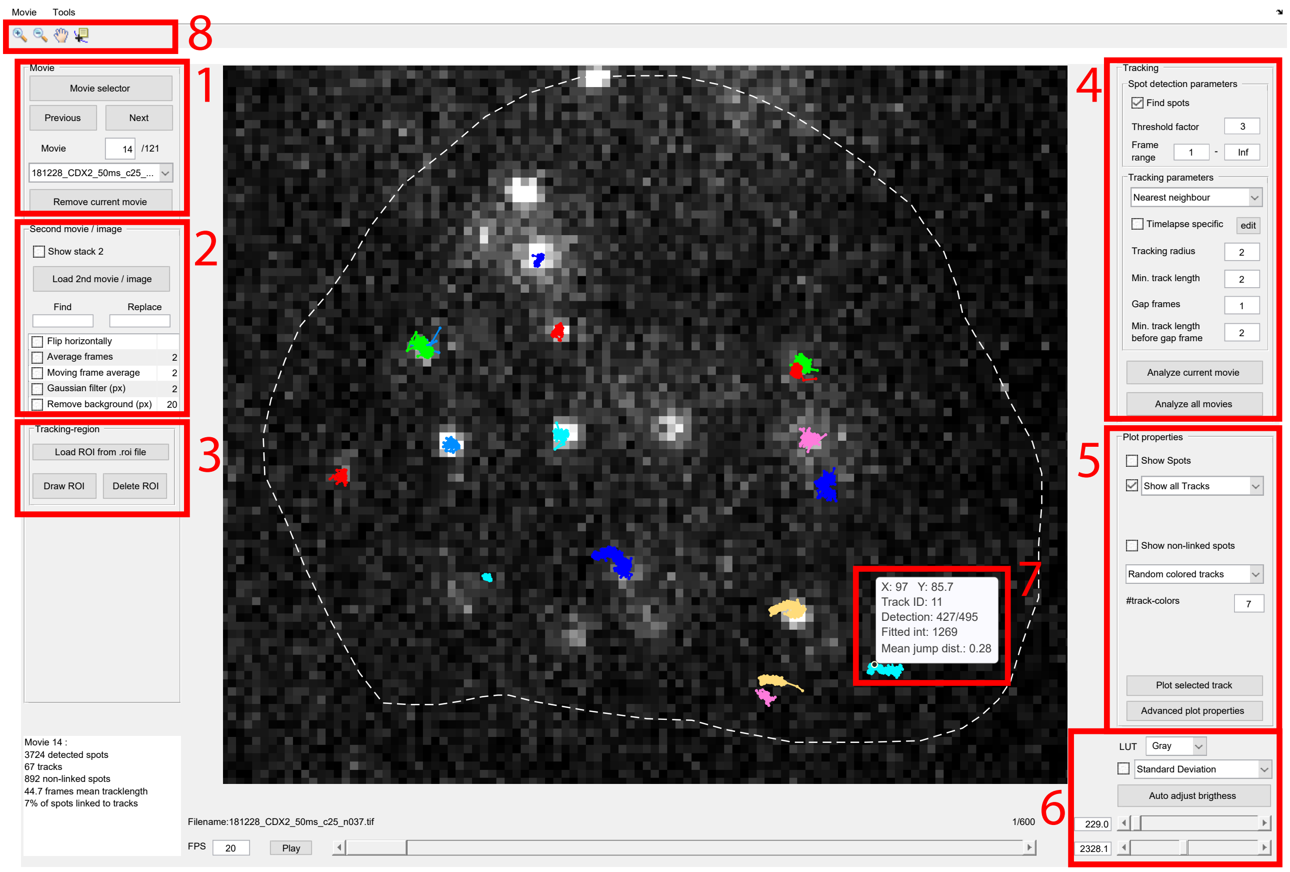

Figure 2.1: Screenshot of the main user interface. 1) Movie panel 2) Second movie / image panel 3) Tracking-region panel 4) Tracking panel 5) Plot properties panel 6) Z-projection and brightness adjustment 7) Toolbar 8) Information on selected track.

1. **Movie panel** Add movies via the "Movie selector" button. Navigate through movies of the current batch with the "next" and "previous" button, by entering a movie number or by selecting a movie from the drop-down list. Single movies can be removed from the current batch with the "Remove current movie" button (the movie is not deleted from your folder).

2. **Second movie/ image panel** Load reference channels carrying information about regions of interest (ROIs) such as the cell nucleus via the "Load 2nd movie / image" button. "Replace" and "Insert" fields help selecting the filename directly without the need of searching through file lists. The reference movie or image can be manipulated with common filtering and averaging operations.

3. **Tracking-region panel** To restrict tracking to a specific region, e.g. the nucleus, a ROI can be drawn or loaded from an existing .roi file which is created automatically every time a ROI is drawn.

4. **Tracking panel** All relevant parameters concerning spot detection and tracking can be set. By clicking "Analyze current movie" or "Analyze all movies" fluorescent molecules are tracked in the current movie or all movies, respectively. To analyze movies with several tracking parameters at once, comma separated values can be entered in the parameter fields. Tracking parameters can also be applied with respect to the corresponding frame-cycle-time of each movie by checking the "Timelase specific" box.

5. **Plot properties panel** All plot related properties such as size, style and coloring of spots and tracks, the range in which tracks are shown and scalebar options are accessible here. Clicking the button "Plot selected track" opens a single track analysis window of the track selected with the "Data tip" in the toolbar. The "track explorer" includes kymographs, intensity, angle and position plot of the track and a mean squared displacement (MSD) analysis (see section 2.6.2).

6. **Z-projection and brightness adjustment** Settings concerning displayed pixels like the color lookup table or brightness and contrast can be adjusted in the lower right corner. Additionally z-projections of the displayed movie or detection and jump distance mappings can be displayed.

7. **Toolbar** Toolbar with tools to zoom in and out, move the plot area and to select specific tracks (see below).

8. **Information on selected track** Selecting a track with the "Data tip" in the toolbar shows basic track information such as track number, current position, fitted intensity and mean jump distance.

2.2 Import movies using movie selector

Clicking the "Movie Selector" Button in the main GUI opens the "Movie selector" window.

Movies can be added in two ways:

1. **Add movies button** .tiff files can be selected directly.

2. **Search folder button** All .tiff files in a selected folder will be added. Optionally, a search string can be entered before clicking "search folder", so that only filenames containing the user specified string are added.

Automatic recognition of frame cycle times
The software is able to recognize the frame cycle times (the time between two consecutive frames) if the filename contains a string consisting of an underline followed by a number and either "ms" (milliseconds), "s" (seconds) or "Hz" (Hertz). For example "_50ms", "_1s" or "_50Hz" (see fig. 2.2). The files will then be grouped according to their frame cycle time. The number of files of each frame cycle time will be displayed together with the time in milliseconds.

Manually set frame cycle times
If no frame cycle time is recognized, the corresponding field will display -1. The time can be manually entered by clicking the frame cycle time field. Movies of different timelapse conditions have to be added separately while entering the timelapse condition for each case.

Set movie order
The user can choose on how the movies should be sorted:

1. Filename: Movies are sorted by their filename

2. Frame cycle time: Movies are sorted according to their frame cycle time (or timelapse condition)

3. String pattern: Movies are sorted by the part of the filename that follows a given string pattern (eg. "_n"), ignoring the part of the filename before the string pattern.

By clicking "OK" the movie selector closes and the first movie will be loaded and displayed in the main GUI. Now is a good time to save the workflow for the first time by clicking "File" » "Save batch file as".

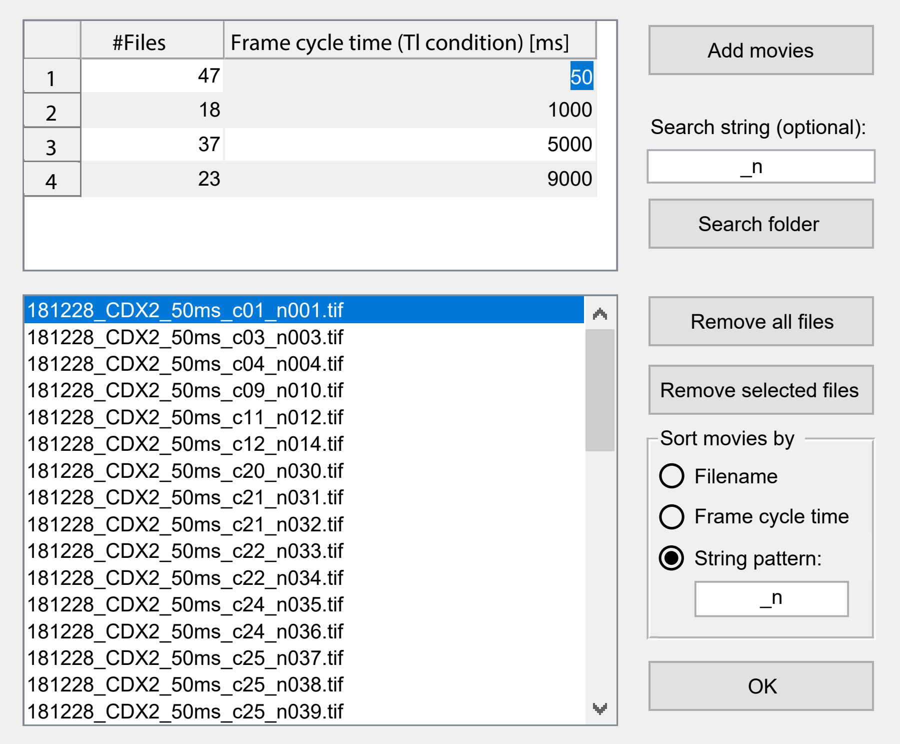

Figure 2.2: Screenshot of transcription factor CDX2 data-set displayed in the movie selector. The search string "_n" was used in combination with the search folder function to add all files in a folder containing that string. The software automatically recognized the frame cycle times (timelapse conditions) as given by the filename.

2.3 Load reference channel

The tracking channel can be overlaid with a second .tiff movie or image (e.g. brightfield image, DAPI or membrane marker). This can be useful for drawing a region of interest (ROI) or localization of proteins with respect to cellular compartments.
A reference channel can be loaded by pressing "Load 2nd movie / image".

In order to ease a laborious search through lists of filenames, a replacement function is implemented. This can be used if the tracking movie and the reference channel share a similar filename for example "cell1_488nm_n001.tif" and "cell1_561nm_n001.tif" and the same folder. The string to replace must be written in the field depicted with "Find", likewise the string which should be inserted must be written in the field depicted with "Replace" (see fig. 2.3). When "Load 2nd movie / image" is pressed the desired filename is directly inserted in the file selection dialog and can easily be opened by hitting "Open".

For the reference channel, following post-processing steps can be applied:

- Flip horizontally: Useful for two color-experiments where a second detection path is split-

of by a beam-splitter.

- Average frames: The second movie is grouped and averaged in frame packages given by a user specified number. The amount of frames is reduced by a factor equal to this number
- Moving frame average: The second movie is averaged by calculating a moving average of the frames. Each frame is built by averaging over a user specified amount of frames before and after each frame. The amount of frames therefore stays the same. The window size is automatically truncated at the beginning and the end of the movie.
- Gaussian filter: Uses Matlabs "imgaussfilt" function to apply a 2-D Gaussian image smoothing filter with a standard deviation (width of the filter kernel) specified by the user (in pixels).
- Remove Background: Subtracts a morphologically opened image from the original image. The radius of the disk element used for the opening operation can be specified by the user (in pixels).

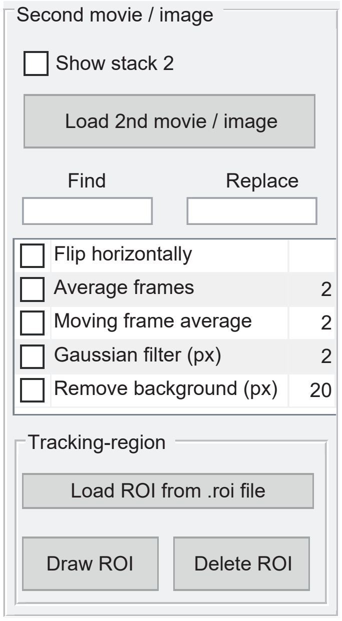

Figure 2.3: Screenshot of the reference channel and ROI panel.

2.4 Region of interest

If desired, a region of interest (ROI) can be drawn. Spot detection will then be restricted to this specific area. If no ROI is drawn, the whole image will be used for detection and tracking.

Directly after drawing is finished, the software will save the ROI in a separate .roi file in a folder called "ROI" located in the same folder as the original movie. Whenever movies are selected via the "Movie selector", the software will check whether a .roi file exists for this file and will load it automatically. .roi files can also be loaded by clicking "Load ROI from .roi file".

2.5 Tracking

Before tracking is started, several parameters can be adjusted in the "Tracking" panel (see fig. 2.4).

2.5.1 Spot detection and tracking parameters

Threshold factor A user defined threshold factor is used to calculate an automatic threshold for spot detection. The threshold is calculated for each movie separately providing comparable detection thresholds between all movies. Reliable detection of single fluorophores is commonly achieved with values between 1 and 5. Movies with a good single-molecule signal (i.e. high signal-to-noise ratio) can usually be analyzed with higher threshold factors.

Frame range Specifies the frames between which the spots should be detected and tracked

Tracking algorithm Ideally the concentration of fluorescent molecules is low enough so that each spot is spatially well separated from others at all times. In this case the nearest-neighbor delivers fast and reliable tracking results. For higher concentrations the u-Track algorithm might provide better results. For spot density considerations see section 2.7.4, Avg. #spots per frame.

Tracking radius Maximum distance at which spots are linked between two consecutive time points. The tracking radius can also be entered in microns by right clicking onto the tracking radius field and selecting "convert from µm". In case of several different frame cycle times as used eg. in time-lapse experiments, consistent tracking radii can be predicted automatically, see 2.5.3.

Min. track length Minimum number of frames a fluorescent molecule has to persist to be accepted as a track.

Gap frames Number of frames a fluorescent molecule can disappear or stay undetected so that tracking is still continued and the fluorescent molecule is combined into a single track.

Min. track length before gap frame Number of frames a track has to exist before closing of gaps (gap frames) is allowed. This can help to prevent connecting random detections in overly crowded movies or movies with bad signal-to-noise ratio (SNR).

Timelapse specific tracking parameters Increasing movement of cellular compartments (eg. chromatin diffusion) with longer frame cycle times, makes it necessary to chose less restrictive tracking parameters. Therefore, above described tracking parameters can be set dependent on their frame cycle time (timelapse condition) (see fig. 2.5).

In some cases it might be desired to try out several combinations of tracking parameters. This can be achieved by inserting comma separated values into the fields of above described parameters. The software will run through all combinations of tracking parameters and save one batch file per parameter set to a folder specified by the user.

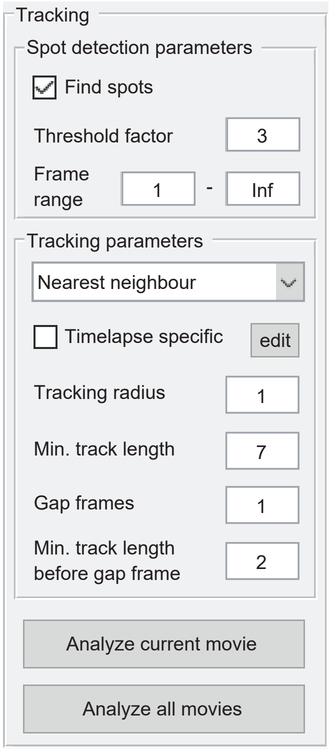

Figure 2.4: Screenshot spot detection and tracking parameter panel.

Timelapse dependent tracking parameters

	TL condition (ms)	Tracking radius	Min. track length	Gap Frames	Min. length before gap frame
1	50	1.2000	4	1	2
2	1000	1.5000	2	1	2
3	5000	1.7000	2	1	2
4	9000	2.1000	2	1	2

Figure 2.5: Screenshot of the timelapse specific tracking parameters window where a different set of tracking parameters is used for each of the four timelapse conditions.

2.5.2 Tracking routine

By clicking either "Analyze current movie" or "Analyze all movies" tracking is performed with the predefined parameters. The tracking routine consists of four steps:

1. **Filtering raw data** Movies are filtered using a wavelet filter as described in [7].

2. **Detecting candidates** First a threshold is applied to the wavelet filtered image to separate signal from background noise. The threshold is calculated by multiplying a user defined threshold factor with the standard deviation of the background noise. Candidate detection is based on local maxima finding at the pixel level. In brief, all pixels that have the same value before and after image dilation are selected.

3. **Position refinement** Candidate positions are refined to a sub-pixel precision by fitting a Gaussian function using the freely available psfFit_Image.m from the TrackNTrace software as described in [14]. We included a maximum distance allowed for spot refinement through gaussian fitting. If the distance between candidate and refined position is larger than 2 pixels, the fit is discarded and the position is refined using the center of mass inside a window of 5 pixels centered at the candidate position. This usually happens if two detections are too close together for correctly fitting a single peak. For spots laying closer together than 2 pixels, the smaller peak is discarded after refinement in order to avoid multiple detections per spot.

4. **Tracking** Linking spots in time and space can be carried out using either a fast nearest-neighbor algorithm or the more sophisticated u-Track algorithm of [9].

2.5.3 Automatic determination of tracking parameters

Click "Tools" » "Predict tracking radii" to open a separate window for tracking radii prediction. The table gives an overview of all parameters. Before running the program, choose wheter

gap-frames shall be allowed (=1) or not (=0). Furthermore the shortest track length can be adapted. Continue by clicking "execute". Fill in the inverse of the trageted track length, i.e. for a targeted mean tracklength of 100, fill in 1e-2. Once the program is done, the table will be updated with the new tracking radii.

2.6 Visualization of tracks

2.6.1 Plotting options

Show spots Shows all detected spots in the current frame

Plotting tracks

- Show tracks in Range: Tracks are visible up to the displayed frame number. The parameter "#frames track is visible" defines how long tracks are plotted after the end of the track. A value of 0 will only show tracks visible in the current frame, for "inf" all tracks are plotted until the current frame.
- Show all Tracks: All tracks of the current movie are plotted
- Show initial positions: Only the position of first appearance of a track will be plotted

Show non-linked spots All positions of non-linked detections are marked with yellow dots.

Coloring of tracks

- Random colored: The track color for each track is chosen randomly and the amount of colors can be entered in the field "#track-colors".
- Colored by track length: Matlabs "parula" color map is used to plot tracks with a color corresponding to their length. Shortest tracks are colored blue while longest tracks are colored in yellow.
- Colored by track length regime: The track colors are chosen according to user defined track length classes. Tracks with a minimum duration of the value entered in the field *min. length to count as long track* are plotted green whereas shorter tracks are shown in red.
- Colored by mean jump distance: Matlabs "parula" color map is used to plot tracks with a color corresponding to their mean jump distance. Tracks with the lowest mean jump distance are colored blue while tracks with the highest mean jump distance are colored in yellow.

Advanced plot properties Clicking the "Advanced plot properties" button opens a window where further adjustments can be made regarding the appearance of the plotted tracks

and spots like the line width and size of the tracks or the style, size and color of the spot marker. A scale bar can be made visible by setting its position, size and text.

2.6.2 Track explorer

Single tracks can be selected via the *Data Tips* function located in the toolbar of the main GUI. A click on "Plot selected track" in the main GUI opens the track explorer showing a visualization and analysis of the selected track. Alternatively the track explorer can be opened via "Tools" » "Track explorer". The results can either be displayed in units of pixels and frames (see fig. 2.6) or microns and seconds (see fig. 2.7) by selecting the corresponding entry in the "Units" panel. Switching between different tracks can be directly done by clicking the "Previous" or "Next" button or by entering a number in the "TrackID" field.

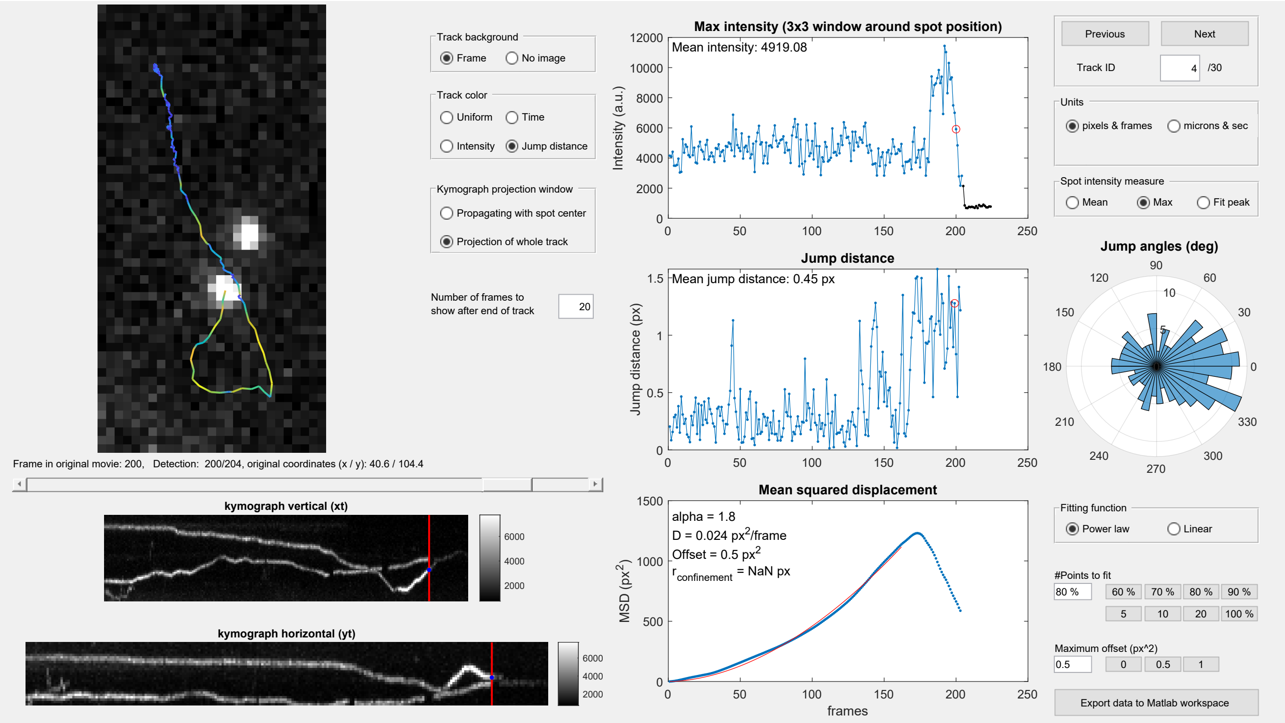

Figure 2.6: Track explorer showing a track of a moving nuclear Myosin VI molecule [3]. The coloring of the track indicates the jump distance from frame to frame which can also be seen in the jump distance plot. Vertical and horizontal kymographs show the movement of the molecule as a projection of one of the spatial dimensions over time. The currently viewed frame in the track plot can be selected with a slider and is indicated in the kymograph as a red bar. A blue dot inside the red bar further indicates the position of the track in the kymographs. Additional plots show the spot intensity and jump distance over time, the mean squared displacement including power law fit and a jump angle histogram. The abundant population of angles between 330° − 30° in the angle histogram and an alpha value of 1.8 are characteristic for a linear motion.

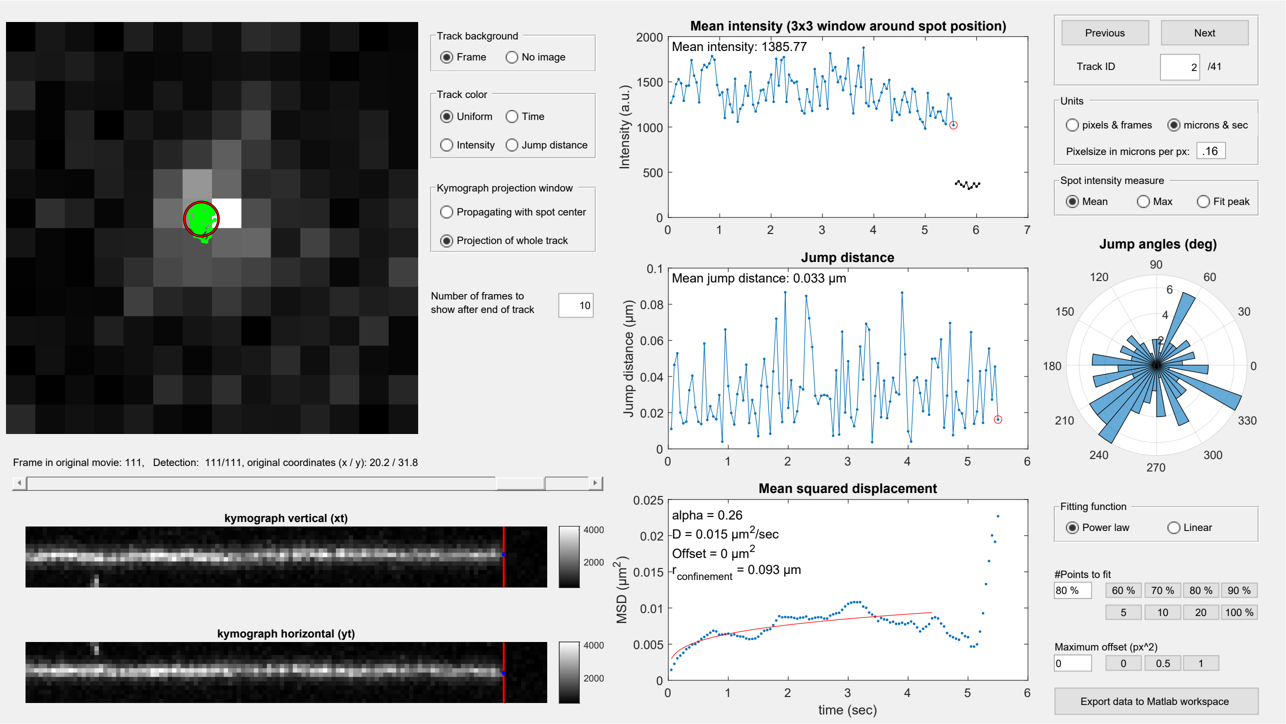

Figure 2.7: Track explorer showing a track of a binding CDX2 transcription factor molecule. In addition to the track, a red circle is plotted with its center at the mean track position and a radius equal to the confinement radius. The confinement radius is calculated by fitting the mean MSD with a confined diffusion model. An angular histogram mostly populated with values between $120° - 240°$ and an alpha value of 0.26 are characteristic for the confined nature of the molecule.

Position plot The selected track is plotted in the upper left corner of the figure. The background image of the track can be selected in the "Track background" panel and can be switched between showing a cutout of the original movie frame or to be dark. The track segments can be colored in four different styles which can be set in the "Track color" panel:

- Uniform: track is uniformly colored in green (see fig. 2.7).
- Time: Matlabs "parula" color map is used to plot track segments with a color corresponding to their time of persistence. The first segments of the track are colored in blue and with increasing time the segments are plotted from light blue over green to yellow.
- Intensity: Matlabs "parula" color map is used to plot track segments with a color corresponding to the intensity of the detected spot. The detection with lowest intensity is colored blue whereas the detection with highest intensity is colored yellow.
- Jump distance: Matlabs "parula" color map is used to plot track segments with a color corresponding to the jump distances between the spots of the track in subse-

quent frames (see fig. 2.6). The minimum jump distance between two subsequent frames is colored blue and with increasing distances the segments are colored from light blue over green to yellow.

Kymographs Horizontal (XT, y-axis projected) and vertical kymographs (YT, X-axis projected) of the track are shown in the bottom left corner of the figure. Here only one dimension is plotted at each timepoint while the other dimension is maximum projected. There are two types of projection windows which can be chosen in the panel "Kymograph projection window":

- Propagating with spot center: The maximum projection is performed within a window of 13 pixels around the spot center. The projection window therefore "travels" with the spot center leading to a kymograph where the spot position is always lying on a horizontal line in the center of the kymograph image.
- Projection of whole track: The maximum projection is performed within the window seen in the position plot on the top left corner. Here the window is kept static so that a movement of the spot results in a movement in the kymograph image.

Kymographs can be continued for additional frames after the track ended by entering a positive number in the field "Number of frames to show after end of track". If "Propagation with spot center" is selected the kymograph projection window is kept at the position of the last spot in the track.

Intensity plot Shows the intensity of the detected spot for each frame of the track. The intensity measure can be chosen in the panel "Spot intensity measure" with the following options:

- Mean: Shows the mean intensity inside a 3x3 pixel window around the spot center for each frame of the track.
- Max: Shows the maximum intensity inside a 3x3 pixel window around the spot center for each frame of the track.
- Fit peak: Shows the peak amplitude of the fitted 2D gaussian function in each frame of the track.

If a number > 0 is entered in the field "Number of frames to show after end of track" the plot is continued after the track ended (drawn in black). In this case the window in which the intensity is determined is held constant at the position of the last spot of the track and is applicable only for the mean and maximum intensity measure. This can be used to check whether the fluorescent molecule has bleached or if the track was lost during tracking process.

Jump distance plot Shows the distances between spots of subsequent frames which were connected to a track.

Angular histogram Displays a polar histogram of jump angles where the jump angle is defined as the change in direction between each step of a track. See also section 2.7.2.

Mean squared displacement plot Shows the mean squared displacement (MSD) of the track. The MSD can either be fitted with a power law or a linear function. The fitted function is plotted on top of the MSD and the fitted parameters are shown in the top left corner of the plot. If a power law is chosen as fit function, an additional fit is used to calculate the confinement radius $r_{\text{confinement}}$ with a confined diffusion model. If the fit was successful, the resulting confinement radius is written below the other fit results and a circle with radius $r = r_{\text{confinement}}$ centered at the mean track position is drawn into the position plot (see fig. 2.7). If a confinement radius could not be determined the result shows "NaN". For more information regarding the MSD analysis see section 2.7.2.

Export to Matlab workspace By clicking this button all results visible in the track explorer are exported to the Matlab workspace.

2.6.3 Detection mapping and jump distance mapping

Detected spots fit to sub-pixel precision with a 2D Gaussian fit are used to create a super-resolved heat map of localizations. Here, fitted positions of all spots (also spots which are not connected to tracks) are accumulated in a 2D histogram. The pixel values therefore correspond to the amount of detections in each pixel (see fig. 2.8 c).
Similar to localization mapping, a heat map of jump distances can be created. For all jumps within a track, a virtual line is drawn between the start and end position of a jump. Each pixel touching this line is assigned with the corresponding jump distance (see fig. 2.8 b). The resulting 2D histogram is then normalized by the amount of events in each pixel to obtain the average jump distance in each pixel.
For both kinds of heat maps the bin size can be chosen as a multiple of the original pixel size resulting in an up –or down-scaled image and can be entered in the field "Scaling factor".

2.7 Data analysis tool

The data analysis tool can be accessed via "Analysis" » "Tracking data analysis.

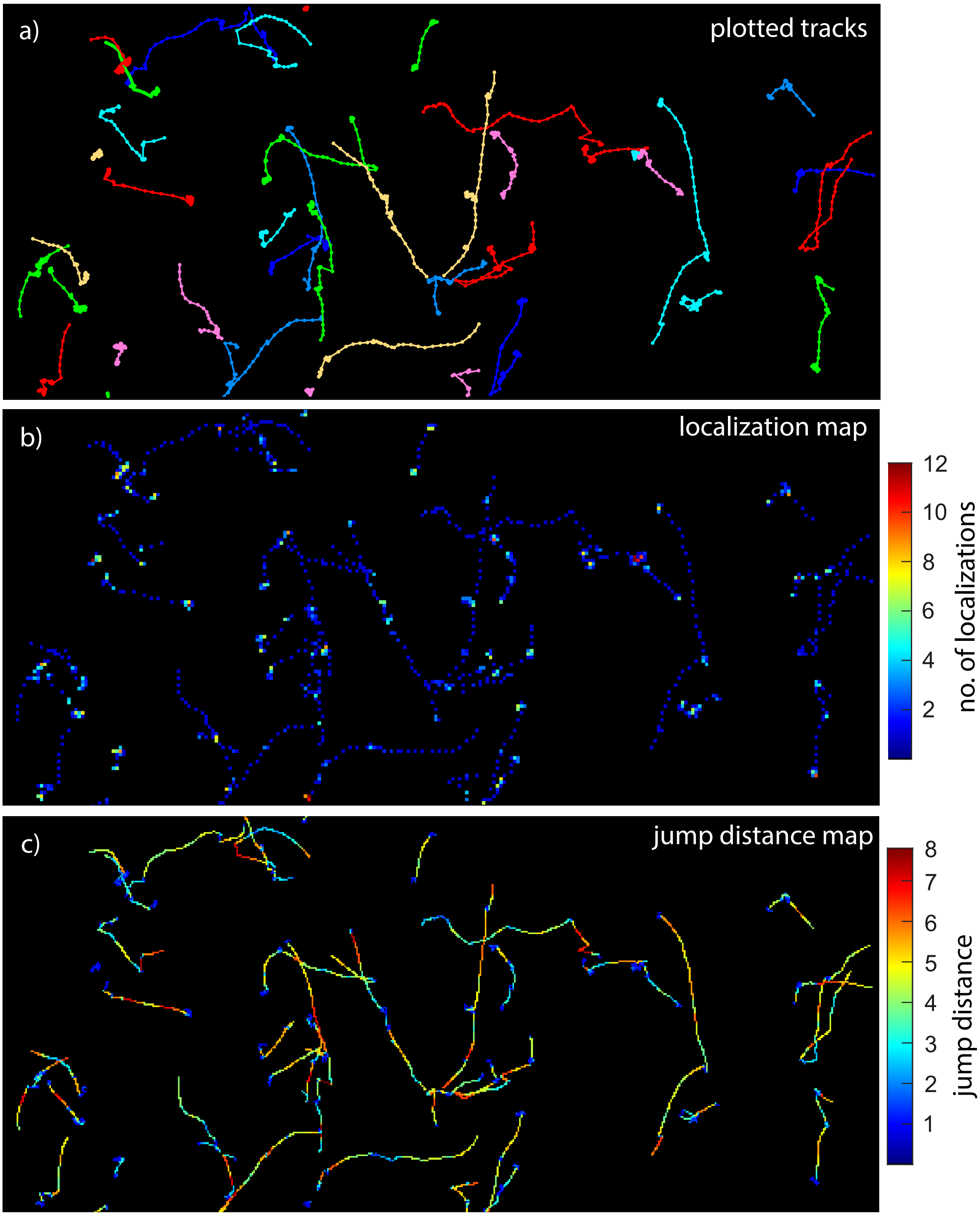

Figure 2.8: a) Plotted tracks of particles from a simulated single molecule movie [1] exhibiting a mixture of confined and linear motion. b) Localization map of detections showing blue pixels where a low number of molecules are localized whereas higher number of localizations per pixels are shown in green, yellow and red. c) Jump distance map of the track particles where blue pixels indicate regions of confined motion whereas pixels which are passed with high jump distances are colored in green, yellow and red.

2.7.1 Overview

1. Batches analyzed and saved with the TrackIt software can be loaded into the data analysis tool via the button "Load batch .mat file(s)". The window below shows a list of loaded batch files where batches to be included in the analysis can be selected. Multiple batches can be selected using the "Ctrl" key.

2. The frame-cycle time selection window in the central left lists all frame-cycle times that are involved in the selected batches and can be used to select subsets of movies. Either a single movie, or a certain frame-cycle time or all movies can be selected to be included into the analysis.

3. The parameter selection window lists all the analysis parameters which are grouped into three tabs: mobility, tracked fractions and statistics. The selected parameter is then displayed in the central plot.

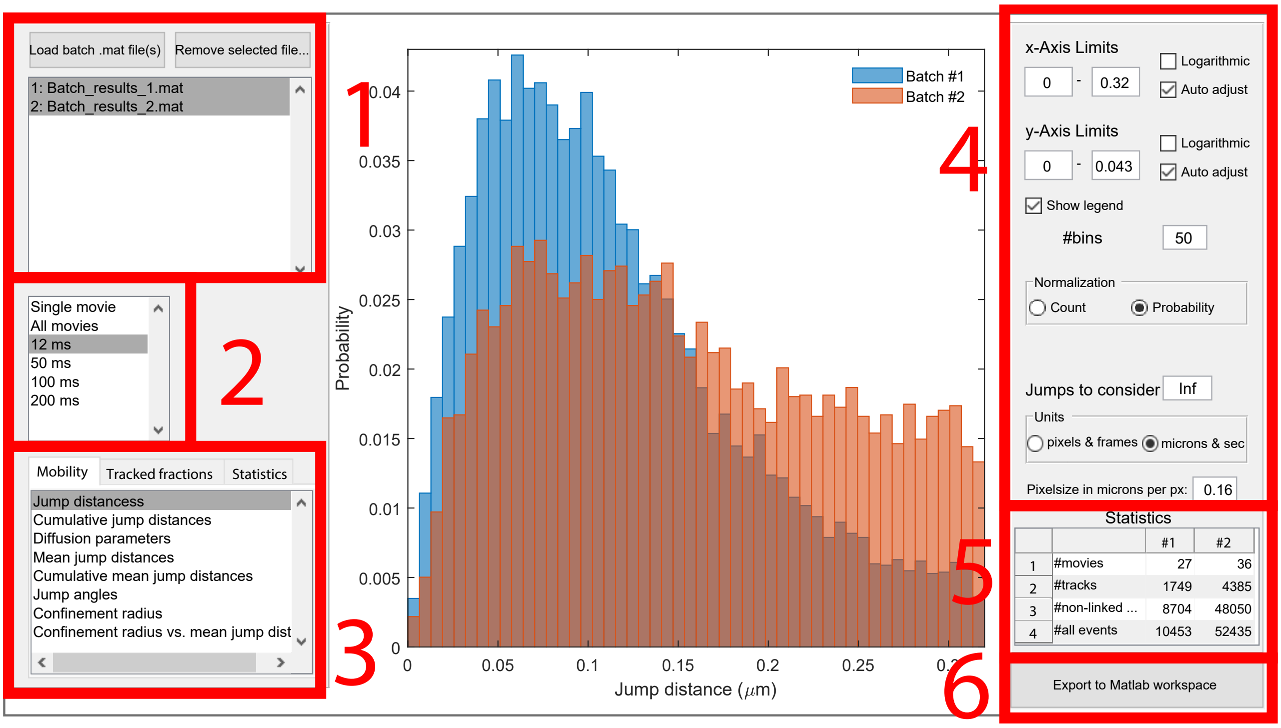

Figure 2.9: Screenshot of the track analysis tool. 1) Batch selection 2) Frame-cycle-time selection 3) Analysis parameter selection window 4) Plotting and analysis options 5) Statistics overview of loaded batches 6) Export to Matlab workspace.

4. Shows a number of plotting options and settings regarding calculations of the different parameters:

   - #bins: Amount of bins in a histogram.

- Jumps to consider: The maximum number of jumps that are considered in each track. This can be useful to prevent an over representation of slow diffusing or bound molecules in the histogram.
- Normalization: Histogram normalization can be switched between "Count" and "Probability". Bin heights are either given by the number of events in each bin or normalized so that the height of all bins sums up to 1, respectively.
- Remove jumps over gap frames: Angles and jump distances extracted from jumps involving gap frame(s) are not considered if the checkbox is checked.
- Units: Can be switched between "pixels & frames" and "microns & sec". If "pixels & frames" is selected, all results concerning length scales are given in the unit of pixels and all results concerning temporal units are in the unit of frames. If "microns & sec" is selected, a conversion factor for the size of one pixel in μm must be entered. All results are then displayed in micrometers using this conversion factor and seconds using the frame-cycle time stored for each movie (displayed in the frame-cycle time window).
- Normalize by ROI-size: Most of the statistical evaluations can be normalized by the size of the ROI in each movie in order to give information on densities (eg. spot densities).
- Illumination pattern: Can be switched between "periodic" and "ITM" (interlaced time-lapse microscopy). ITM is an illumination scheme where two subsequent image acquisitions are followed by a dark time. This scheme is specifically designed to gather quantitative information on chromatin-bound fractions and proportions of stable bound molecules [13]. Due to a non-periodic image acquisition, the illumination scheme has to be taken into account when calculating the number of dark periods that an immobile molecule survived (see Section 2.7.3).

5. The statistics overview windows gives information on movie numbers and molecule counts for each loaded batch.

6. Exports two variables to the Matlab workspace: "currentPlotValues" contains the data which is shown in the current plot, "allHistogramResults" contains all the raw data that is used to display the different parameters implemented in the histogram tool.

2.7.2 Mobility analysis tab

Jump distances Displays the distribution of jump distances in a histogram. The jump distance is defined as the Euclidean distance between the positions of two linked spots of a track in consecutive frames.

Cumulative jump distances Displays the cumulative distribution of squared jump distances. This distribution can be used to fit either a 2-rate or 3-rate diffusion model (see below). The fitted diffusion functions can be visualized by selecting "show 1-rate diffusion fit", "show 2-rate diffusion fit" and "show 3-rate diffusion fit".

Diffusion parameters Cumulative distributions of squared jump distances are fitted with one, two or three exponential components corresponding to one, two or three effective diffusion constants D_{1-2} or D_{1-3} with amplitudes A_{1-2} or A_{1-3}, respectively [2].

If the one-rate model is selected in the pop-up menu, the following fit function is applied

$$f_1(X) = \left(1 - e^{-\frac{X}{D_1}}\right) / \left(e^{-\frac{C_1}{D_1}} - e^{-\frac{C_2}{D_1}}\right) \tag{2.1}$$

If the two-rate model is selected in the pop-up menu, the following fit function is applied

$$f_2(X) = A_1 \left(1 - e^{-\frac{X}{D_1}}\right) + (1 - A_1) \left(e^{-\frac{C_1}{D_2}} - e^{-\frac{X}{D_2}}\right) / \left(e^{-\frac{C_1}{D_2}} - e^{-\frac{C_2}{D_2}}\right) \tag{2.2}$$

If the three-rate model is selected in the pop-up menu, the applied fit function is

$$\begin{aligned} f_3(X) = &A_1 \left(1 - e^{-\frac{X}{D_1}}\right) + A_2 \left(1 - e^{-\frac{X}{D_2}}\right) \\ &+ (1 - A_1 - A_2) \left(e^{-\frac{C_1}{D_3}} - e^{-\frac{X}{D_3}}\right) / \left(e^{-\frac{C_1}{D_3}} - e^{-\frac{C_2}{D_3}}\right) \end{aligned} \tag{2.3}$$

Functions are normalized to account for the lower and upper limit of jump distances $C_1 = 0$ and $C_2 = d_{max}$ where d_{max} is the tracking radius. Error bars in the plot indicate 95% confidence intervals of the fitted diffusion constants and amplitudes. To ensure that the fit converges to a global minimum, diffusion constant start values can be entered on the right side of the figure. For a visualization and control of the fitted function see *cumulative jump distances* above.

Important: fitting distributions populated with jump distances from mixed frame-cycle times will lead to wrong results!

Mean jump distances Shows a histogram of the mean jump distances of all tracks. The mean jump distance $\bar{d}$ of a track is defined as:

$$\bar{d} = \frac{1}{n}\sum_{i=1}^{n} d_i \tag{2.4}$$

where n is the number of jumps in a track and d_i are the jump distances of the track.

Cumulative mean jump distances Displays a cumulative histogram of the mean jump distances.

Jump angles Displays a polar histogram of jump angles which are defined as the change in direction between each step of a track. The jump angle histogram can give additional information about diffusion properties or directional behavior. A circular polar histogram may indicate a random, brownian motion. A polar histogram where bins are concentrated around 180° indicates either a confined diffusion or a bound state (where the apparent jump distance due to a localization imprecision is higher than the fluorescent molecule movement). Tracks with its polar histogram centered near 0° tend to have only small directional changes and may indicate a directed motion. See also [8] and [4].

Diffusion constants from MSD fit Shows the distribution of diffusion constants which are extracted by either fitting the the mean squared displacement (MSD) with a linear function

$$MSD = 4 \cdot D \cdot t + \text{offset} \tag{2.5}$$

or with a power law

$$MSD = 4 \cdot D \cdot t^{\alpha} + \text{offset} \tag{2.6}$$

where D is the diffusion coefficient and α a coefficient that indicates the motion type. For the offset a maximum allowed value can be set in the field "Max. offset".

The MSD is calculated as

$$MSD(\tau) = \frac{1}{\tau}\sum_{t=1}^{\tau}(x(t) - x(t+\tau))^2 + (y(t) - y(t+\tau))^2 \tag{2.7}$$

where x(t) and y(t) are the coordinates of the spot within a track at the time t. τ is the maximum time interval between two spots of a track and can be set in the field "#Points to fit". Here, either a numeric value can be entered or a percentage value (eg. 90%). Entering a percentage value between 60% and 90% can be useful as the statics that goes into each point in the MSD gets worse with τ reaching $\tau_{\text{max}} = t_{\text{track}} - 1$ where t_{track} is the track duration. A minimum track length can be entered in the field "shortest track".

Alpha values from MSD fit Shows the distribution of alpha-values which are extracted by fitting the MSD (see above).

Confinement radius Shows the distribution of confinement radii calculated by fitting the MSD with a confined diffusion model [10]

$$MSD_{circle} = R^2 \cdot \left(1 - e^{-4 \cdot D \frac{t}{R^2}}\right) + \text{offset} \tag{2.8}$$

where R is the radius of confinement and D is the local diffusion coefficient. The offset is introduced to account for the finite localization precision. To use exclusively tracks which show a confined motion, tracks with alpha-values above a threshold specified in the field "upper alpha limit" are discarded.

Confinement radius vs. mean jump distance Displays a 2-dimensional plot where the mean jump distance of a track is plotted versus its confinement radius (see above) [10]. This representation can give insights into different mobility classes of single molecules.

2.7.3 Tracked fraction analysis tab

Bound fractions (Bf) Bound fractions can either be analyzed from movies with continuous or interlaced illumination. The illumination pattern can be selected in the "Illumination pattern" button group. The fractions of molecules belonging to two different binding time classes can be approximated using the interlaced time-lapse microscopy (ITM) scheme [6, 13]. In ITM, two frame acquisitions separated by a short dark time are followed by a longer dark time. This illumination scheme allows to classify molecules by their binding time. Molecules count as long bound (N_{long}) if they survive at least one long dark time, short bound (N_{short}) if the molecule survives only one short dark time and diffusive if the molecule is detected in only one frame ($N_{\text{non-linked}}$). The ITM scheme can be used for a bound fraction analysis by choosing the option "Interlaced (ITM)" in the field "Illumination pattern". Choosing "Continuous", bound fractions can also be calculated from continuous movies, but come with high errors and have to be handled with care.

Generally, accurate bound fractions can only be obtained if bleaching is corrected [13]. Three different types of bound fractions can be extracted:

Tracks vs. all events Shows the fraction of all bound molecules with respect to all counted events

$$\mathrm{Bf}_{\text{all bound}} = \frac{N_{\text{bound}}}{N_{\text{all events}}} = \frac{N_{\text{long}} + N_{\text{short}}}{N_{\text{long}} + N_{\text{short}} + N_{\text{non-linked}}} \tag{2.9}$$

where N_{bound} is the number of all molecules classified as bound and $N_{\text{all events}}$ is the number of all events.

Long tracks vs. all events Shows the fraction of long bound molecules with respect to all counted events

$$\mathrm{Bf}_{\text{long vs. all}} = \frac{N_{\text{long}}}{N_{\text{all events}}} = \frac{N_{\text{long}}}{N_{\text{long}} + N_{\text{short}} + N_{\text{non-linked}}} \tag{2.10}$$

where $N_{\text{all events}}$ is the number of all events.

Long tracks vs. long + short tracks Shows the fraction of long bound molecules with respect to all bound molecules

$$\mathrm{Bf}_{\text{long bound}} = \frac{N_{\text{long}}}{N_{\text{bound}}} = \frac{N_{\text{long}}}{N_{\text{long}} + N_{\text{short}}} \tag{2.11}$$

Pooled and movie-wise values Bound fractions are determined for each movie (displayed as blue triangles) and can show a significant variance between cells or movies. The average of these movie-wise values is calculated and displayed as a red dot. To avoid an over-representation of outliers or movies with low molecule counts, events for each binding time class can be summed up over all movies resulting in a single "pooled" bound fraction of all movies which is displayed as a black dot.

Error estimation The error for the movie-wise bound fraction (red error bar) is given by the standard error of the mean

$$\Delta_{\mathrm{BF}_{\text{moviewise}}} = \frac{\mathrm{stdev}(\mathrm{BF}_{\text{moviewise}})}{\sqrt{N_{\text{movies}}}} \tag{2.12}$$

where stdev($BF_{moviewise}$) is the standard deviation of the movie-wise bound fraction values and N_{movies} is the amount of movies for which bound fractions are calculated.

The error for the pooled bound fraction (black error bar) is calculated through linear error propagation

$$\Delta_{BF_{pooled}} = \frac{1}{N_{denominator}} \cdot \delta_{N_{numerator}} + \frac{N_{numerator}}{N^2_{denominator}} \cdot \delta_{N_{denominator}} \tag{2.13}$$

where $N_{numerator}$ is either N_{long} or N_{bound} and $N_{denominator}$ is either N_{bound} or $N_{all\ events}$. The errors δ are estimated by the counting errors with $\delta_{N_{numerator}} = \sqrt{N_{numerator}}$ and $\delta_{N_{denominator}} = \sqrt{N_{denominator}}$.

No. of tracks Shows the total number of tracked molecules in each movie.

No. of non-linked detections Shows the number of non-linked detections. This can either be single detections that have not been linked to tracks or detections of a track with a track length shorter than the minimum track length set by the user (see fig. 2.1 panel 4).

No. of all events (tracks + non-linked) Shows the number of total events in each movie calculated by the total number of tracks plus the number of non-linked detections.

No. of long tracks In the case of continuous illumination the amount of tracks which are longer than the threshold entered in the field "count as long track if track is longer than" is shown. If the interlaced illumination pattern (ITM) is selected, the displayed values are given by the amount of tracks which survive a specific number of long dark periods entered in the field "#survived dark periods to count as long track".

No. of short tracks Shows the amount of short tracks in each movie which is given by the total number of tracks minus the number of long tracks (see above).

2.7.4 Statistics tab

Track lengths Histogram showing the lengths of tracks. The track length is defined as the amount of frames a track survives. The minimum length of a track is therefore two.

Avg. track length Shows the average track length in each movie.

Avg. no. tracks per frame The average number of tracks per frame in a movie is calculated by counting the number of tracks visible in each frame divided by the number of frames in the corresponding movie.

Avg. no. spots per frame Shows the average number of spots per frame in each movie calculated by dividing the total amount of spots by the number of frames in the movie. If "Normalize by ROI-size" is selected, the values are divided by the size of the ROI resulting in the average spot density per frame. Based on experience, the average spot density should be below $2.5 \cdot 10^{-3} \frac{\text{spots}}{\text{px} \cdot \text{frame}}$ for the nearest neighbor algorithm to link trajectories correctly.

ROI size Shows the size of the region of interest (ROI) of each movie either in μm^2 or in number of pixels depending on the selected units.

2.8 Analysis of dissociation rates with GRID

Via "Analysis" » "Analyse dissociation rates (GRID)" tracking results can be directly analyzed using the GRID software. Therefor track lengths (in seconds) are passed over to the GRID tool. For a detailed description of GRID please read the GRID user manual or refer to [12]. GRID is fully compatible with batch files created with the TrackIt software and can be loaded directly from the GRID GUI via "Files" » "Load File". GRID is available under `https://gitlab.com/GebhardtLab/GRID`.

2.9 Data export and movie creation

Export all data to Matlab workspace

By selecting "File" » "Export all data to Matlab workspace" in the menu bar of the main GUI, the data structure is saved in a variable of type "struct" with the name "currentBatch" in the Matlab workspace.

Export tracks

An export dialog window (see fig. 2.10) can be opened by clicking "File" » "Export tracks to .mat or .csv". In the "Compatibility" popup-menu .mat files can be chosen to be compatible either with Spot-On [5] or with vbSPT [11]. Only .csv files are compatible with Spot-On. Spot-On is available under `https://spoton.berkeley.edu/` and vbSPT under `https://github.com/bmelinden/vbSPT`.

Movie creation

A movie inlcuding all selected plot elements can be created by clicking "File" » "Create .avi movie". A .avi movie file will be created using the exact same plot properties as currently

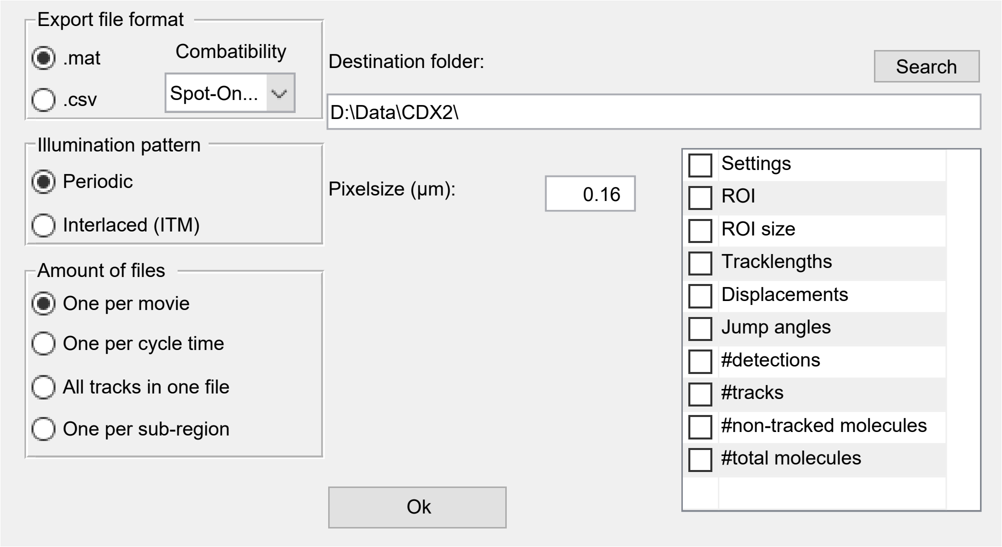

Figure 2.10: Track export window.

shown in the main GUI. The playback speed (frames-per-second, FPS) can be set in the "FPS" field at the lower part of the main GUI.

2.10 Additional tools

2.10.1 Spot statistics

A tool showing information about the detected spots can be open via "Analysis" » "Spot statistics" where the following data can be shown and retrieved:

Peak spot intensity Shows a histogram of the peak intensity of all detected spots. The peak intensity is defined as the highest pixel value of all pixels within a radius of 2 px around the spot center.

Fitted spot intensity Shows a histogram of the fitted intensities of all detected spots as given by the maximum of the 2D Gaussian fit.

Spot SNR Histogram of the signal-to-noise ratio (SNR) of all detected spots. The SNR is calculated in a square window with side length of 17 pixels around the spot peak position at the center pixel (see fig. 2.12) as follows:

$$\mathrm{SNR} = \frac{\overline{I}_{\mathrm{blue}} - \overline{I}_{\mathrm{white}}}{\sigma_{\mathrm{white}}} \tag{2.14}$$

where $\overline{I}_{\mathrm{blue}}$ and $\overline{I}_{\mathrm{white}}$ is the mean intensity of the pixel values of the respective area and σ_{white} denotes the standard deviation of the the pixel values of the white area.

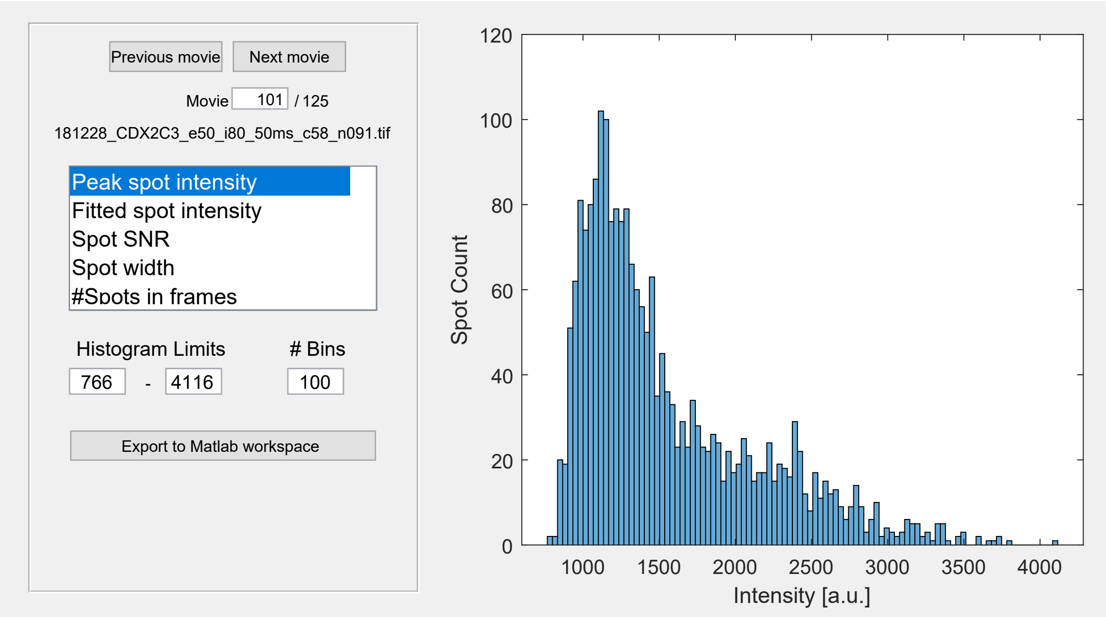

Figure 2.11: Spot statistics tool.

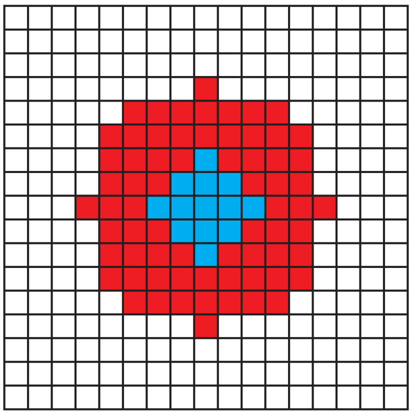

Figure 2.12: Pixel mask used for calculating the spot signal-to-noise ratio (SNR). **White**: Area where the mean background signal $\overline{I}_{\text{white}}$ and standard deviation of background noise σ_{white} are calculated. **Blue**: Area where the mean intensity $\overline{I}_{\text{blue}}$ of the spot is calculated. **Red**: Not used for calculation

Spot width Histogram of the spot width of all detected spots as given by the standard deviation σ of the Gaussian fit.

#spots in frames Shows a plot of the number of detected spots in each frame.

2.10.2 Kymograph

The kymograph tool can be started by clicking "Tools" » "Kymograph". As soon as a rectangular region is drawn the kymograph opens (see fig. 2.13). The cutout region of the original movie is shown in the upper left part and the current frame can be chosen using the slider. Horizontal (XT, Y-axis projected) and vertical kymographs (YT, X-axes projected) of the region are shown in the lower part of the figure. Here only one dimension is plotted at each time point while the other dimension is maximum projected. A red bar indicates the currently shown movie frame. In the top right part of the figure a plot shows the maximum pixel value in each frame of the kymograph. The amount of frames shown in the kymograph and the intensity plot can be entered in the field "No. of frames shown in kymograph and intensity plot". A click on "Export to Matlab workspace" the original movie cutout, the intensity and kymographs are exported to the Matlab workspace.

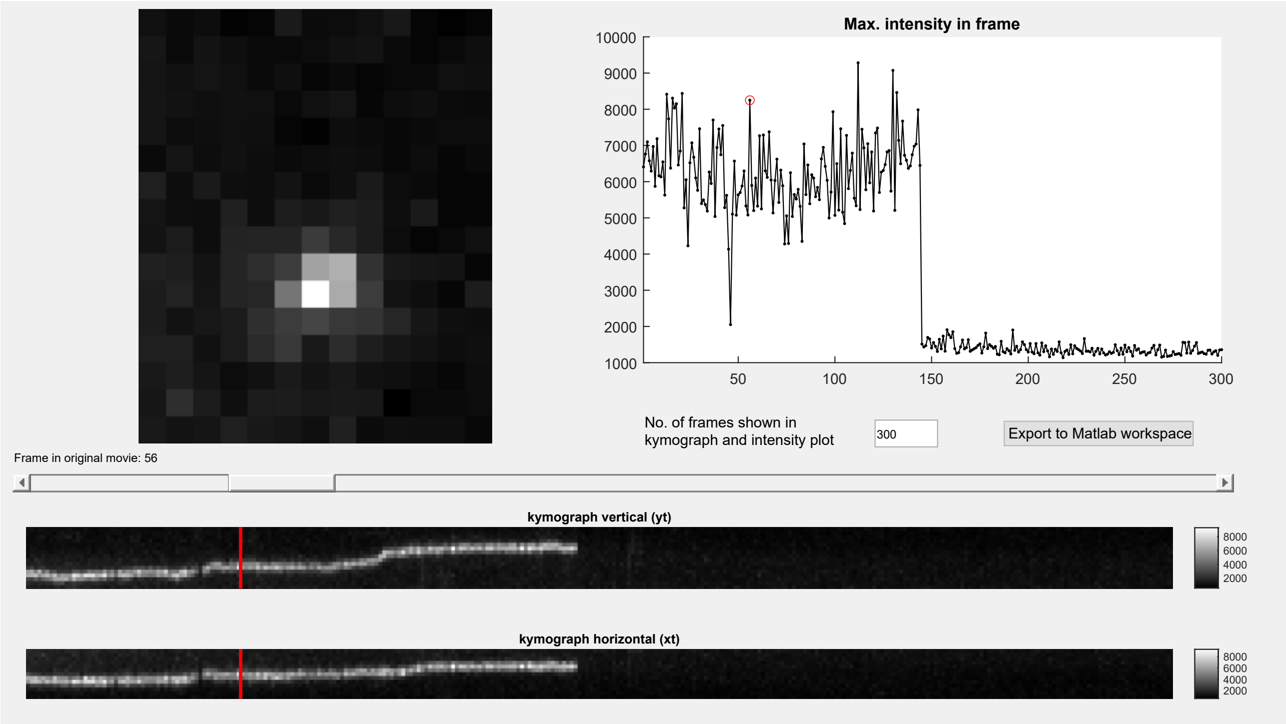

Figure 2.13: Kymograph tool. Top left: cutout of the original movie frame. Bottom: horizontal and vertical kymographs with red bars indicating the currently viewed frame. Top right: maximum pixel value of each frame in the kymograph plotted versus the frame number.

2.10.3 Movie splitter

Movies containing dark frames or movies containing more than one channel, as commonly originated from multiple-color experiments, can be split using the "Movie splitter" tool located in the "Tools" menu bar (see fig. 2.14). The amount of splits as well as the number of frames in each sequence can be defined by the user. A name entered in the field "Add-on to original filename" will be added to the original filename for each splitted movie part. If desired a .txt file containing the .tiff metadata can be created for each original movie. TrackIt uses the Bio-Formats library (`https://www.openmicroscopy.org/bio-formats/`) to extract metadata.

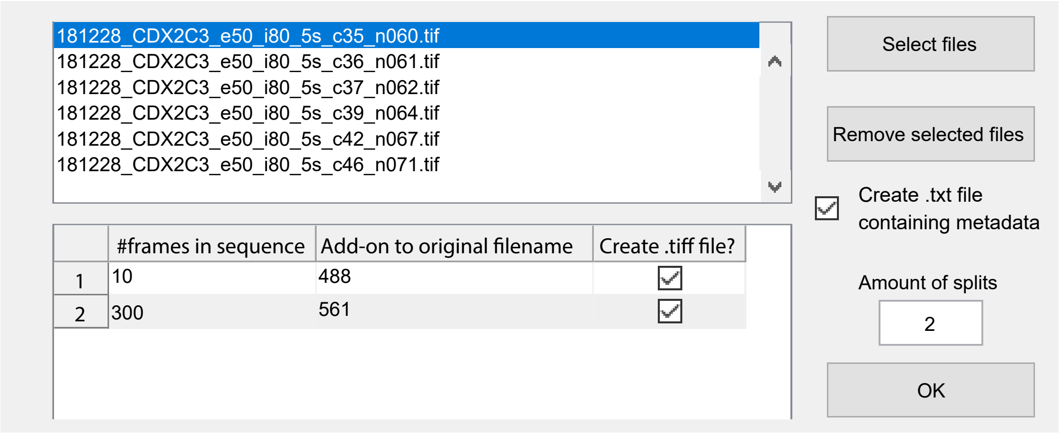

Figure 2.14: Movie splitter tool.

3 Benchmark

3.1 Tracking performance

In this section the reliability and performance of the TrackIt tracking routine is evaluated using simulated single molecule movies from a particle tracking challenge [1]. The image data plus a stand-alone software for performance evaluation is publicly available under `http://www.bioimageanalysis.org/track/index.php#datac`. Tracking and timing performances are evaluated using the datasets "Vesicles" and "Receptors" and a comparison is made between TrackIt and 14 other software tools which participated in the particle tracking challenge.
The "Vesicles" dataset shows particles diffusing in a two-dimensional plane simulated with a Brownian motion model. The "Receptor" dataset also shows particles simulated in a two-dimensional plane where the movement can switch between confined and linear motion. Both datasets were simulated with three different densities (100, 500 and 1000 molecules per frame, or 0.0004, 0.0019 and 0.0038 molecules per pixel and frame respectively) at four different signal-to-noise ratios (1, 2, 4 and 7), summing up to a total of 12 simulation movies per dataset.
Two different performance measures were considered for assessing the tracking accuracy and comparing the results with other software tools:

alpha value indicates to which extent estimated tracks and ground truth tracks overlap: $\alpha(X, Y) = 1 - d(X, Y)/d(X, \emptyset)$ where $d(X, Y)$ denotes the total distance between a set of estimated tracks X and a set of paired ground truth tracks Y. $\emptyset$ denotes a set of dummy tracks so that $d(X, \emptyset)$ is the maximum possible total distance from the ground truth. The value of α therefore ranges from 0 to 1 (perfect match) and does not penalize nonpaired tracks.

beta value additionally penalizes estimated tracks where no corresponding ground truth tracks where found (nonpaired tracks): $\beta(X, Y) = (d(X, \emptyset) - d(X, Y))/d(X, \emptyset) + d(\bar{Y}, \emptyset))$ where $\bar{Y}$ is the set of nonpaired tracks, and $d(\bar{Y}, \emptyset)$ is the penalty score. It takes the value α if no nonpaired tracks exist and converges to zero with higher number of nonpaired tracks.

For more information on the tracking performance measures, please refer to [1].

Figure 3.1 shows the performance results of the participants of the particle tracking challenge plotted together with the results of our TrackIt software using the nearest neighbor algorithm.

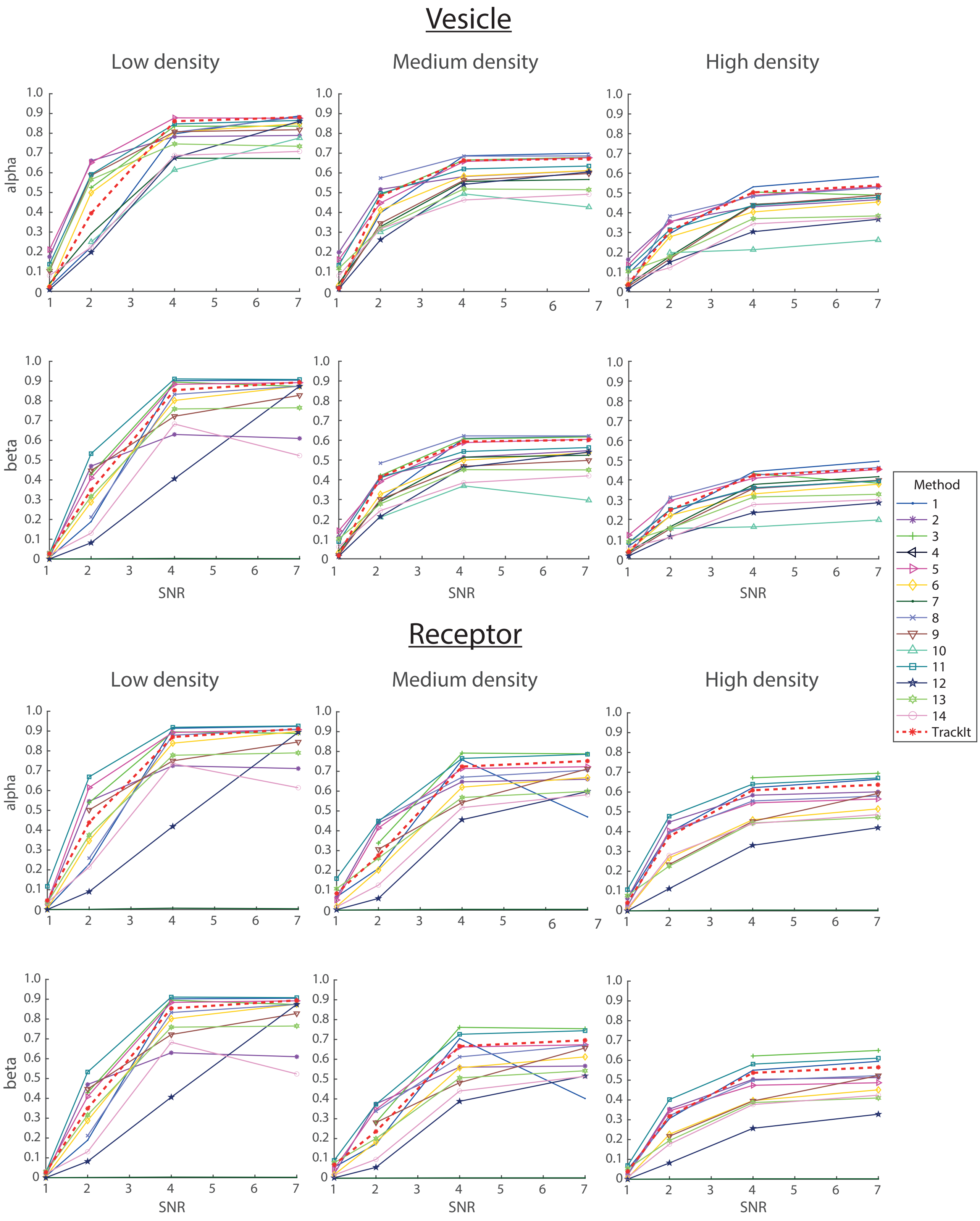

Figure 3.1: Tracking performance of participants of the particle tracking challenge [1] and TrackIt in the two scenarios "Vesicle" and "Receptor". Values of the performance measures α and β are plotted as a function of SNR for three different densities (low, medium and high). Tracking with the TrackIt software was performed using the nearest neighbour algorithm.

For low densities and medium to high signal-to-noise ratios (4 and 7), TrackIt delivers reliable, above average tracking results. With lower SNR and higher density the performance drops as expected. TrackIt is not designed to work for very low SNR or high single molecule densities. Still it is evident that all the methods that participated in the particle tracking challenge show a drastically reduced tracking performance for low SNR (2 and 1) and for medium to high particle densities. In order to obtain reliable tracking results we recommend to use particle densities below 0.0025 molecules per pixel and frame and a SNR above 3.

3.2 Computation times

An overview of the computation times of each method for the two scenarios are shown in table 3.1. Computation times for Method 1-14 are taken from [1] where tracking was performed on a workstation with a 64-bit Intel Xeon X5550 processor (2.67 GHz), 24 GB RAM and Windows 7 operating system. Tracking with the TrackIt software using the nearest neighbor algorithm was performed on a workstation with a 64-bit Intel Core i5-6500 processor (3.2 GHz), 16 GB RAM and Windows 10 operating system. Although tracking was performed on different workstations, it is evident that TrackIt is in a class with the fastest participating softwares in the particle tracking challenge.

**Scenario**							**Receptor**					
**Density**		**Low**				**Mid**				**High**		
**SNR**	**1**	**2**	**4**	**7**	**1**	**2**	**4**	**7**	**1**	**2**	**4**	**7**
**Method 1**	13	13	13	13	15	15	14	60	19	36	30	26
**Method 2**	21	10	8	9	402	149	141	119	1433	995	499	439
**Method 3**	-	33	25	29	-	58	113	109	-	-	356	344
**Method 4**	-	-	-	-	-	-	-	-	-	-	-	-
**Method 5**	206	205	62	67	468	320	274	256	948	636	819	705
**Method 6**	688	1097	541	529	945	1681	1340	1219	1020	3043	2414	2274
**Method 7**	4948	3556	2371	2313	6405	5609	4719	4186	5817	11438	9260	9796
**Method 8**	-	319	364	383	-	6449	2925	2719	43375	9505	9873	9545
**Method 9**	-	14	11	14	-	19	26	39	-	29	39	74
**Method 10**	-	-	-	-	-	-	-	-	-	-	-	-
**Method 11**	94	10	8	8	270	76	117	104	2081	3117	2006	1838
**Method 12**	164	162	153	158	161	167	168	147	163	207	167	156
**Method 13**	25	19	9	9	65	45	17	18	97	44	31	29
**Method 14**	839	592	45	45	819	2706	103	106	4150	10931	503	399
**TrackIt**	5	3	3	2	7	6	9	8	6	35	52	48

**Scenario**							**Vesicles**					
**Density**		**Low**				**Mid**				**High**		
**SNR**	**1**	**2**	**4**	**7**	**1**	**2**	**4**	**7**	**1**	**2**	**4**	**7**
**Method 1**	13	13	10	13	13	21	16	16	39	49	43	39
**Method 2**	21	14	12	12	410	406	231	201	2229	1998	991	733
**Method 3**	-	34	32	38	-	126	157	165	-	-	418	425
**Method 4**	-	-	-	-	-	-	-	-	-	-	-	-
**Method 5**	69	20	17	19	90	111	133	136	177	326	402	387
**Method 6**	842	703	743	702	1569	4119	2554	2626	2334	6578	5244	5490
**Method 7**	7012	3028	2388	2396	8282	6015	7716	19879	9335	11203	19644	21980
**Method 8**	-	-	622	632	-	5240	4714	4310	10279	13214	16802	15479
**Method 9**	12	14	13	17	12	25	42	45	11	33	104	122
**Method 10**	-	46	46	40	-	103	116	112	-	119	193	179
**Method 11**	107	11	10	9	282	604	408	430	3348	7113	10854	9135
**Method 12**	57	56	289	134	51	58	156	149	59	77	252	156
**Method 13**	25	17	11	10	51	20	20	20	80	33	38	38
**Method 14**	869	220	37	26	4287	495	134	126	2773	1118	475	311
**TrackIt**	5	4	3	3	4	18	16	16	5	59	104	96

Table 3.1: Timing in seconds for all particle tracking methods for the scenarios "Receptor" and "Vesicle".

3.3 Diffusion coefficient analysis

In order to validate our diffusion coefficient analysis routine we simulated 50 videos each containing one molecule diffusing in a two-dimensional plane switching between two diffusion states. The corresponding diffusion coefficients were $D_1 = 2\ \mu m^2/s$ and $D_2 = 20\ \mu m^2/s$. The dwell time before switching was set to 1 second for both states implying equally distributed fractions of states $F_1 = 0.5$ and $F_2 = 0.5$. The movies had a window size of 150 x 150 pixels with a pixel size of 160 nm/px and a length of 500 frames with a frame cycle time of 10 ms. After tracking with TrackIt we fit the cumulative density distribution of squared displacements with a Brownian diffusion model with two diffusion components (see section (2.7.2)). We additionally estimated the precision of the diffusion constants and fractions by performing a resampling (not part of TrackIt) where we fit 500 subsets of the jump distance distribution each containing 80% of randomly drawn jump distances out of the full dataset and calculated the standard deviation of the results. We further analyzed the tracking results with Spot-On [5] and vbSPT [11], to which the tracks can be directly exported from TrackIt (see section (2.9)). The results in Table 3.2 show that all three methods produce similar results close to the ground truth values. The overall fitting error Spot-On delivered from its Matlab version was 9.7e-05. Please note that the choice of the right diffusion analysis software is always dependent on the type of data but also on individual requirements and shall not be discussed here.

	$\mathbf{D_1}$(µm²/s)	$\mathbf{D_2}$(µm²/s)	$\mathbf{F_1}$	$\mathbf{F_2}$	**Loc. err.** (µm)
**Ground truth**	2.00	20.00	0.500	0.500	-
**TrackIt**	2.32 ± 0.03	19.98 ± 0.13	0.434 ± 0.004	0.566 ± 0.004	-
**vbSPT**	2.37 ± 0.03	20.09 ± 0.93	0.439	0.561	-
**Spot-On**	2.11	19.60	0.440	0.560	0.045

Table 3.2: Diffusion analysis results including diffusion constants $D_{1,2}$ and fractions of molecules in each state $F_{1,2}$. Spot-On additionally fits the localization error.

Bibliography

[1] N. Chenouard, I. Smal, F. De Chaumont, M. Maška, I. F. Sbalzarini, Y. Gong, J. Cardinale, C. Carthel, S. Coraluppi, M. Winter, A. R. Cohen, W. J. Godinez, K. Rohr, Y. Kalaidzidis, L. Liang, J. Duncan, H. Shen, Y. Xu, K. E. Magnusson, J. Jaldén, H. M. Blau, P. Paul-Gilloteaux, P. Roudot, C. Kervrann, F. Waharte, J. Y. Tinevez, S. L. Shorte, J. Willemse, K. Celler, G. P. Van Wezel, H. W. Dan, Y. S. Tsai, C. O. De Solórzano, J. C. Olivo-Marin, and E. Meijering. Objective comparison of particle tracking methods. *Nature Methods*, 11(3):281–289, 2014.

[2] J. C. M. Gebhardt, D. M. Suter, R. Roy, Z. W. Zhao, A. R. Chapman, S. Basu, T. Maniatis, and X. S. Xie. Single-molecule imaging of transcription factor binding to DNA in live mammalian cells. *Nature Methods*, 10(5):421–426, 2013.

[3] A. Große-Berkenbusch, J. Hettich, T. Kuhn, N. Fili, A. W. Cook, Y. Hari-Gupta, A. Palmer, L. Streit, P. J. Ellis, C. P. Toseland, and J. C. M. Gebhardt. Myosin vi moves on nuclear actin filaments and supports long-range chromatin rearrangements. *bioRxiv*, 2020.

[4] A. S. Hansen, A. Amitai, C. Cattoglio, R. Tjian, and X. Darzacq. Guided nuclear exploration increases CTCF target search efficiency. *Nature Chemical Biology*, 16(3):257–266, 2020.

[5] A. S. Hansen, M. Woringer, J. B. Grimm, L. D. Lavis, R. Tjian, and X. Darzacq. Robust model-based analysis of single-particle tracking experiments with spot-on. *eLife*, 7:1–33, 2018.

[6] L. Hipp, J. Beer, O. Kuchler, M. Reisser, D. Sinske, J. Michaelis, J. C. M. Gebhardt, and B. Knöll. Single-molecule imaging of the transcription factor SRF reveals prolonged chromatin-binding kinetics upon cell stimulation. *Proceedings of the National Academy of Sciences of the United States of America*, 116(3):880–889, 2019.

[7] I. Izeddin, J. Boulanger, V. Racine, C. Specht, A. Kechkar, D. Nair, A. Triller, D. Choquet, M. Dahan, and J. Sibarita. Wavelet analysis for single molecule localization microscopy. *Optics Express*, 20(3):2081, 2012.

[8] I. Izeddin, V. Récamier, L. Bosanac, I. I. Cissé, L. Boudarene, C. Dugast-Darzacq, F. Proux, O. Bénichou, R. Voituriez, O. Bensaude, M. Dahan, and X. Darzacq. Single-molecule tracking in live cells reveals distinct target-search strategies of transcription factors in the nucleus. *eLife*, 2014(3):1–27, 2014.

[9] K. Jaqaman, D. Loerke, M. Mettlen, H. Kuwata, S. Grinstein, S. L. Schmid, and G. Danuser. Robust single-particle tracking in live-cell time-lapse sequences. *Nature Methods*, 5(8):695–702, 2008.

[10] J. Lerner, P. A. Gomez-Garcia, R. L. McCarthy, Z. Liu, M. Lakadamyali, and K. S. Zaret. Two-Parameter Mobility Assessments Discriminate Diverse Regulatory Factor Behaviors in Chromatin. *Molecular Cell*, 79:1–12, 2020.

[11] F. Persson, M. Lindén, C. Unoson, and J. Elf. Extracting intracellular diffusive states and transition rates from single-molecule tracking data. *Nature Methods*, 10(3):265–269, 2013.

[12] M. Reisser, J. Hettich, T. Kuhn, A. A. P. Popp, A. Große-Berkenbusch, and J. C. M. Gebhardt. Inferring quantity and qualities of superimposed reaction rates from single molecule survival time distributions. *Scientific Reports*, 10(1):1–13, 2020.

[13] M. Reisser, A. Palmer, A. P. Popp, C. Jahn, G. Weidinger, and J. C. M. Gebhardt. Single-molecule imaging correlates decreasing nuclear volume with increasing TF-chromatin associations during zebrafish development. *Nature Communications*, 9:5218, 2018.

[14] S. C. Stein and J. Thiart. TrackNTrace: A simple and extendable open-source framework for developing single-molecule localization and tracking algorithms. *Scientific Reports*, 6(November):1–7, 2016.
